# Regulation of the JAK/STAT signaling pathway in spinal cord injury: an updated review

**DOI:** 10.3389/fimmu.2023.1276445

**Published:** 2023-11-08

**Authors:** Xinyu Guo, Chao Jiang, Zhe Chen, Xiaohui Wang, Fan Hong, Dingjun Hao

**Affiliations:** ^1^ Department of Spine Surgery, Honghui Hospital, Xi'an Jiaotong University, Xi’an, China; ^2^ Department of Developmental Genetics, Max Planck Institute for Heart and Lung Research, Bad Nauheim, Germany

**Keywords:** cytokines, spinal cord injury, the JAK/STAT signaling pathway, neuroinflammation, neural homeostasis

## Abstract

Cytokines are involved in neural homeostasis and pathological processes associated with neuroinflammation after spinal cord injury (SCI). The biological effect of cytokines, including those associated with acute or chronic SCI pathologies, are the result of receptor-mediated signaling through the Janus kinases (JAKs) as well as the signal transducers and activators of transcription (STAT) DNA-binding protein families. Although therapies targeting at cytokines have led to significant changes in the treatment of SCI, they present difficulties in various aspects for the direct use by patients themselves. Several small-molecule inhibitors of JAKs, which may affect multiple pro-inflammatory cytokine-dependent pathways, as well as STATs, are in clinical development for the treatment of SCI. This review describes the current understanding of the JAK-STAT signaling in neuroendocrine homeostasis and diseases, together with the rationale for targeting at this pathway for the treatment of SCI.

## 1 Introduction

With the development of technologies, more and more people are dying due to spinal cord injuries (SCIs) caused by traffic accidents, sports injuries and other accidents, which can cause severe neurological damage and functional impairments for patients (1–6). The characteristics of traumatic SCIs in China from 2009-2018 were retrospectively analyzed in two national reports, whose results showed that the incidence rate increased from 45.1 per million in 2009 to 66.5 per million in 2018, with the highest incidence rate in the eastern part and the fastest growth in the western part of the country; the incidence rate of males was higher than that of females, which was higher in rural population than in urban population (7, 8). According to the incomplete statistics in international epidemiological authoritative journals and the international SCI information network every year, there are various causes of patients with SCIs that are close to 20 million, of which simply in the United States, the proportion of patients with SCIs is close to 1000 (9, 10). What is more alarming is that with the rapid development of social economy as well as the accelerated pace of work and life, the incidence of SCIs has increased by 40 times in the past 20 years, and this number is being constantly updated (11). Traffic accidents are the leading cause of SCIs, accounting for 46.9% of all of them, followed by falls, smashes and crushes, which account for 33.1%.

**Figure 1:**
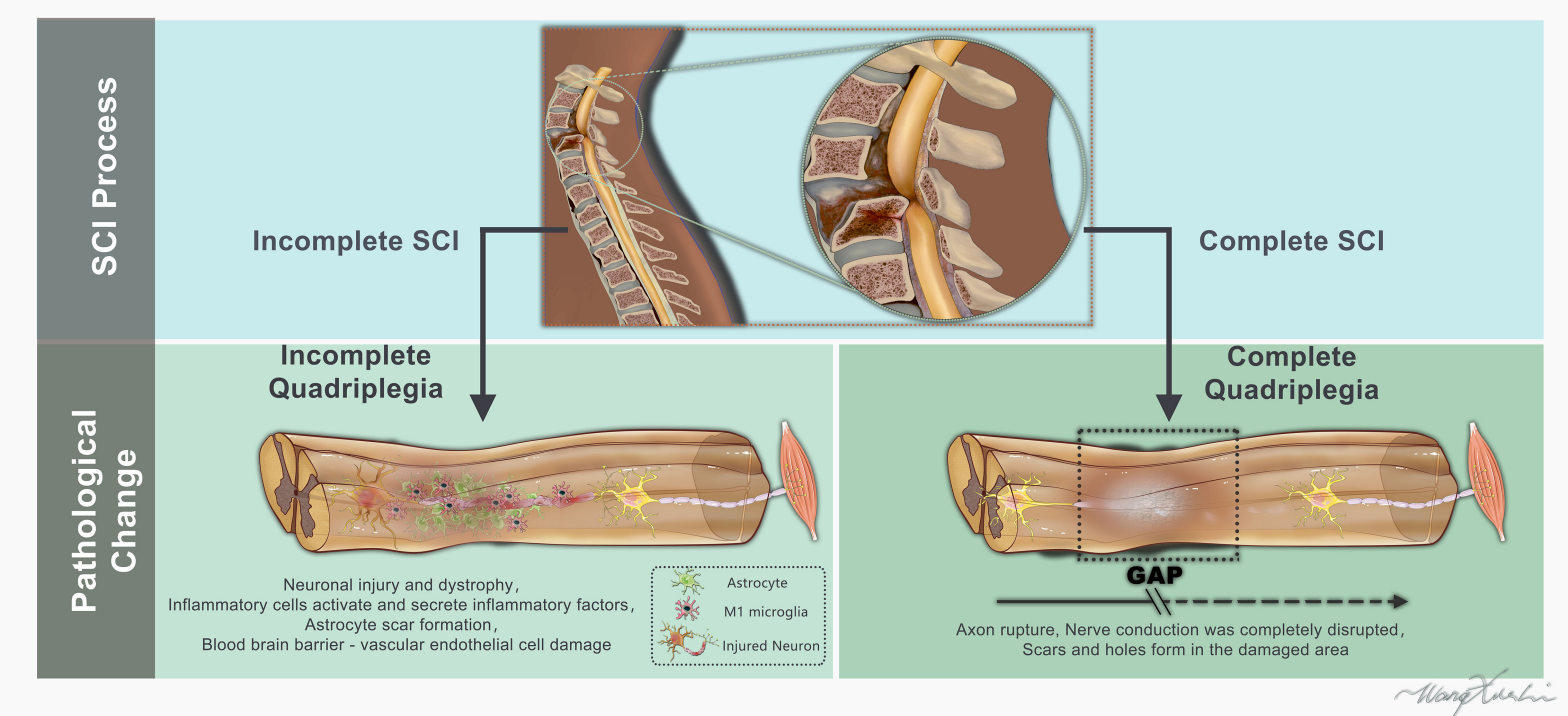
Schematic representation of the human spinal cord and histological features appearing after complete or incomplete spinal cord injury (lesion epicenter, glial scar).

Spinal cord activity is controlled by the brain. Sensory impulses from extremities and the trunk reach the brain through specific pathways in the spinal cord, which are transmitted down to the spinal cord through specific conduction pathways to complete limb movements after a high-level analysis in the brain. SCIs are a common disease in orthopedics and neurosurgery, which are mainly characterized by sensory-motor dysfunctions below the level of SCIs, and most patients are paralyzed in bed and have difficulty walking, which seriously affects their quality of life. Great progress has been made in the development of modern medical technologies, but SCIs are still a major medical problem in clinical diagnosis and treatment, and most patients cannot be cured (12, 13). The primary characteristic of acute SCIs is the mechanical deformation of the spinal cord, followed by acute spinal cord ischemia, then secondary SCIs occur as time passes. The process is affected by ischemia, calcium- and sodium-mediated cellular injuries, cell deaths, apoptosis and inflammation (14, 15). Secondary injuries are a multifactorial, waterfall-amplified cascade of tissue self-destruction that occur after a primary injury, which sometimes exceed that of primary SCIs, resulting in an edematous apoptotic zone that extends from the central hemorrhagic necrosis to a length of 2-3cm adjacent to the spinal cord, which is often replaced by neuroglial scar tissues in later stages (16, 17). SCIs severely affect the motor function of patients, which induce a series of cytokine-mediated inflammatory responses, including tumor necrosis factor (TNF-a), interleukin-6 (IL-6) and IL-B, all of which lead to secondary injuries. Previous studies have shown that the mRNA gene expression of TNF-a, IL-6 and IL-B, etc. is significantly upregulated after SCIs. The activation of tyrosine kinase (JAK) and signal transducers, and the activator of the transcription (STAT) signaling pathway is important for signal transmission from the cell surface to the nucleus, the activation of JAK at the cell membrane leads to the phosphorylation of STAT in the cytoplasm, and signals are transmitted from phosphorylated STAT to the nucleus, followed by the translocation of target genes, which plays an important role in the pathophysiological changes after SCIs (18–23). The JAK/STAT signaling pathway is a fundamental part of the eukaryotic cell cycle, which affects cell growth, survival, development and differentiation (15, 24–28) (**Figure 1**).

The JAK/STAT pathway was originally discovered in the interferon γ signaling system (29–32). Interferon γ, a pro-inflammatory cytokine, has been shown important in controlling phosphorylation and STAT1 nuclear translocation, modulating the expression of major histocompatibility complex (MHC) genes. Interferon γ appears in the cytoplasm of motor sensory neurons 2 days after cutoff and remains in place for 8 days. Currently, the STAT pathway has been identified in mucor, worms, flies and chordates (33–35), but has not been observed in fungi or plants. 7 STAT genes are present in mammals, namely STAT1, STAT2, STAT3, STAT4, STAT5A, STAT5B and STAT6. When cells are not stimulated, STAT proteins are located in the cytoplasm, remaining inactive (36), while when coupled to receptors, they are rapidly activated and then enter the intracellular structural domain of the receptors through the Src homology region 2 (SH2) binding to the phosphorylated complexine residues of the receptors.

Many production factor receptors have tyrosine kinase activity of their own. The tyrosine kinase activity required by such receptors is provided by JAK proteins (37, 38), which are evolutionarily conserved. There are 4 JAK proteins in mammals, namely JAK1, JAK2, JAK3 and Tyk2, which are produced during mutations that signal or inhibit JAK protein activity. JAK proteins interact with receptor binding and JAK tyrosine phosphorylation activation, while the receptors also undergo tyrosine phosphorylation, generating STAT binding sites (39–41). Through the phosphorylation of STAT proteins on tyrosine residues, STAT homodimers and heterodimers are produced, through which STAT dimers are transported from the cytoplasm to the nucleus and bind to DNA.

The activation of the JAK and STAT signaling pathway is an important pathway for signal transmission from the cell surface to the nucleus. Cell membrane JAK activation leads to STAT phosphorylation in the cytoplasm and signal transmission from phosphorylated STAT to the nucleus, followed by target gene translocation, which plays an important role in pathophysiological changes after SCIs. There is a time-dependent activation of the JAK/STAT signaling pathway after SCIs, which is correlated with changes in JAK and STAT expression, so it is important to investigate the JAK/STAT pathway after SCIs. Therefore, the relationship between the JAK/STAT pathway and SCIs is reviewed in this paper.

## 2 The JAK/STAT signaling pathway

**Figure 2:**
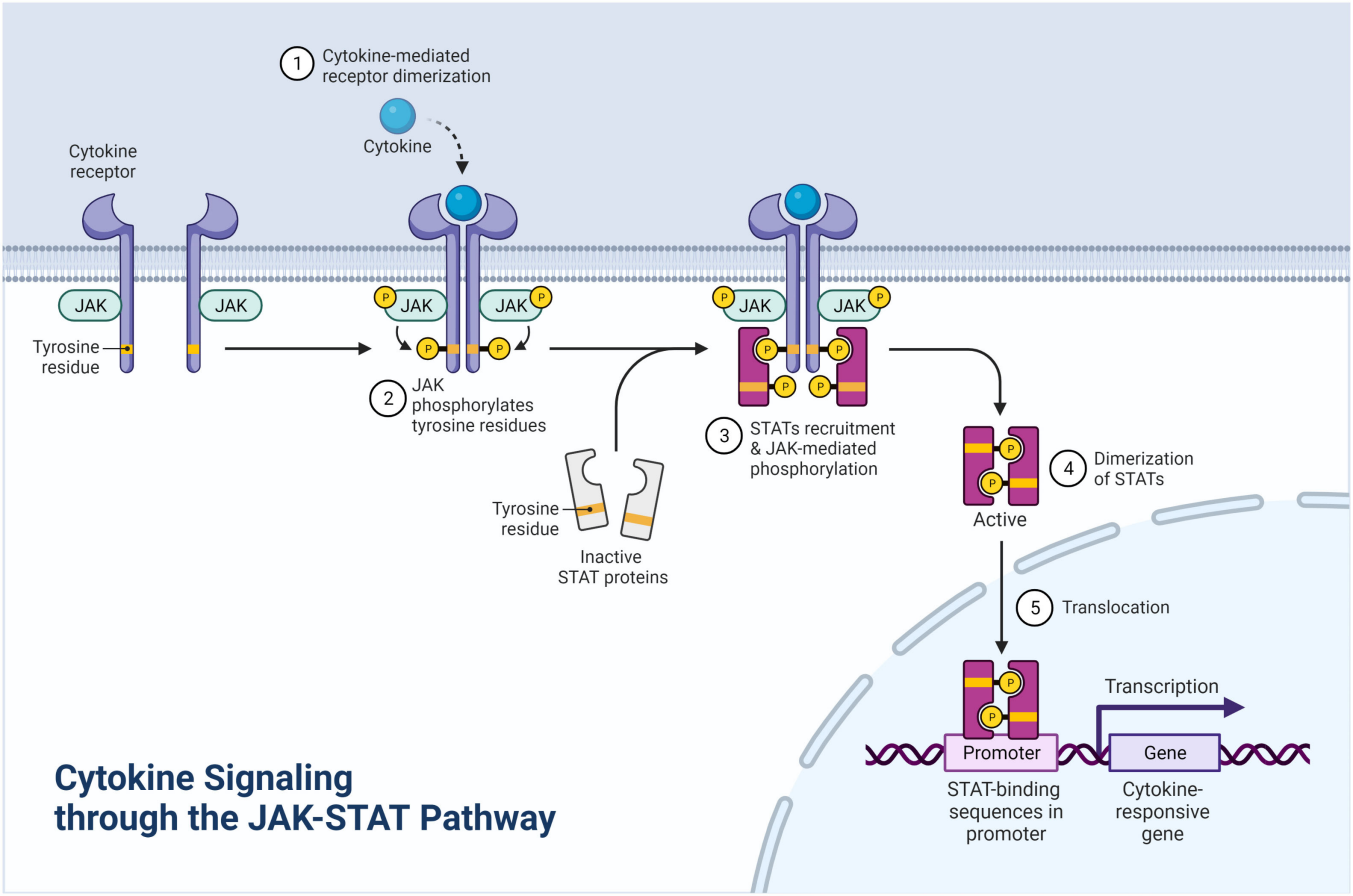
Cytokine signaling through the JAK-STAT pathway (Created with BioRender.com).

The JAK/STAT signaling pathway is an intracellular signal transduction pathway where the JAK and STAT family are activated. The JAK family consists of JAK1, JAK2, JAK3 and TYK2; the STAT family includes STATI, STAT2, STAT3, STAT4, STAT5A, STAT5B and STAT6. The JAK/STAT pathway plays a major role in regulating gene expression, which involves the activation of cell membrane receptors by growth factors, hormones or cytokines, resulting in the activation of JAKs in cell membranes (42–44). The MOET phosphorylation of proteins is an important biochemical pathway controlled by growth factors or cytokines. Initially, JAKs become tyrosine-phosphorylated by binding to membrane receptors (1, 45–47). Subsequently, STATs in the cytoplasm are activated through tyrosine phosphorylation (1, 2, 15, 45), which in turn translocates STAT dimers to the nucleus, where they bind to specific cis-acting elements, followed by the transcription of different target genes (1, 2, 47, 48). Nerve growth and glial scar formation are closely correlated with inhibited the JAK/STAT signaling pathway in SCI areas. After axonal injuries, STAT3 activation and overexpression cause the protection of neurons. It has also been reported in other studies that: STAT3 is activated in reactive astrocytes of the injured areas (15, 49). During intact spinal cord development, STAT3 is mostly found in forefoot motor neurons and dendritic-like structures (50) (**Figure 2**).

### 2.1 A role of the JAK/STAT pathway in the proliferation and differentiation of neural stem cells

The JAK/STAT pathway promotes the regeneration of astrocytes. Bonni et al. (51) found the JAK/STAT pathway critical for glial cell differentiation. Authors have demonstrated that ciliary neurotrophic factor (CNTF) receptors are activated in cortical precursor embryonic cells, followed by the activation of JAK1, STAT1 and STAT3, meanwhile neuronal stem cells and neural progenitor cells are induced to differentiate into astrocytes. STAT3 is also involved in the differentiation of glial cells according to several other studies (25, 41, 51). Moreover, glycoprotein 130 (gp130) is involved in the JAK/STAT signaling pathway that promotes astrocyte differentiation into neural stem cells (NSCs) and neural progenitor cells (NPCs) (52, 53). Sriram et al. (54) similarly demonstrated the critical role played by STAT3 activation via gp130 in inducing reactive astrocytes. Glycoproteins are multichain receptors found on cell membranes, among which there are ligand-binding receptors as well as nonligand-binding membrane glycoproteins, such as gp130 implicated in signal transduction through cytokines such as the interleukin (IL) family (54). Upon binding to the multichain receptor complex, these cytokines induce the dimerization of gp130 molecules through the JAK activation at cell membranes, followed by STAT3 phosphorylation at the Tyr705 site, leading to a translocation to the nucleus. Studies have shown that prolactin (CPRL) is also able to promote astrocyte differentiation and proliferation by phosphorylating JAK2/STAT1 and STAT3. According to some research (55, 56), CPRL stimulates astrocyte proliferation and cytokine production, and the authors found that by increasing JAK2 tyrosine phosphorylation and the subsequent STAT1a as well as STAT5a phosphorylation, CPRL could stimulate astrocyte growth. Additional studies have shown that in mice with a specific STAT3 knockout, astrocyte growth can be inhibited (57).

The JAK/STAT pathway induces the differentiation of neurons and oligodendrocytes. Through the activation of STAT1, STAT3 and STAT5 through JAK phosphorylation, NSCs in adult CNSs express cytokine interleukin (IL), and JAK inhibitors are effective in blocking this activation (58, 59). A number of studies have demonstrated that the inhibition of JAK/STAT signaling pathway proteins is responsible for neuronal differentiation and axonal growth. Inhibitory proteins, like the suppressors of cytokine signaling 2, SOCS3 and SOCS6, function through insulin-like growth factors and growth hormones (GHs), negatively affecting the JAK/STAT signaling pathway (59–61). SOCS2 is expressed in neural stem cells to inhibit GH signaling while promoting neuronal differentiation and neural inhibition (62). SOCS3 deletion promotes axonal regeneration in adult mice after SCIs by promoting the gp130-mediated CNTF signaling pathway through the JAK/STAT pathway (63). Regarding the different isoforms of JAKs, JAK1 is dominant in astrocyte differentiation, while JAK2 is also essential in the proliferation of NSCs (64, 65). Deleted JAK2 inhibits the activation of proto-oncogenes (c-mycs), while JAK2 and STAT5 proteins may be essential for cell proliferation. The inhibition of JAK3 results in the differentiation of NPCs into neurons and dendritic cells (66). In addition, *in vitro* and *in vivo* STAT3 inhibition can promote neural regeneration.

### 2.2 Time-dependent neuroprotective effect of JAK/STAT on reactive astrocytes

Among the many scar formations following CNS injuries, Kernie et al. (67) suggested that they might be the result of newly-generated astrocytes rather than the activation or migration of endogenous astrocytes. A traditional view is that SCIs cause alterations in microenvironmental factors and result in reactive astrocytes that inhibit axonal regeneration, whose main causes include the release of chondroitin sulfate proteoglycan, extracellular matrix-inhibiting molecules and several inflammatory cytokines, including IL-1β, IL-6 and TNF-α. STAT3 phosphorylation after SCIs and the reactive release of these inflammatory cytokines suggest an important role of STAT3 for astrocyte activation (68–71), especially within the region of SCIs. In a study of Okada et al. (72), by inhibiting the IL-6 receptor, not only astrocyte differentiation, but also astrocyte proliferation was prevented, and an axonal regeneration following SCIs was reduced. In a relative sense, analyses of time-dependent reactive astrocytes are crucial for identifying their role in SCIs. During the acute and subacute SCI phase, by re-establishing spinal cord blood flow barriers–astrocytes, whose role in SCIs is to separate the spinal cord lesion sites from healthy spinal cord tissues, prevent greater inflammatory responses, massive cellular degenerative deaths and tissue damage in secondary injuries. Therefore, some scholars suggest that astrocyte proliferation after CNS injuries is dependent on STAT3 activation as a critical step in the formation of glial palsy, which in turn limits the spread of inflammation. It has also been reported that the inhibition of STAT3 after SCIs leads to a reactive migration of astrocytes, which results in an extensive inflammatory cell infiltration, demyelination and a severe loss of the motor function (73, 74). Furthermore, Leung et al. demonstrated that the inhibition of SOCS3 expression prolonged STAT3 expression and activation in reactive astrocytes, thereby improving SCI healing and the motor function of spinal cord.

### 2.3 JAK/STAT signaling pathway produces the neuroprotective effect by modulating the astrocyte secretion of polypeptides

In addition to the neuroprotective effect, astrocytes also secrete polypeptides, which are involved in endogenous neuroprotection and cellular repair. It is believed that reactive astrocytes secrete and influence a number of cytokines that have an impact on their surrounding cells (microglia and neurons) while affecting the astrocytes themselves (75). In response to a SCI, reactive astrocytes may protect neurons and oligodendrocytes, thus preserving the motor function. A possible explanation is that astrocytes secrete peptides (astrocyte-derived cytokines and trophic factors) altering the microenvironment. Cytokines that can act as neurotrophins include IL-1, TNF-α, IL-6, IL-11 and nerve growth factors (NGFs), ciliary neurotrophic factors, basic fibroblast growth factors as well as leukemia inhibitory factors (LIFs) (1, 76). Increasing evidence suggests that factors released by reactive astrocytes may protect injured tissues and cells when mediated through the JAK/STAT signaling pathway (77–80).

A particular focus on IL-6, as well as LIF, CNTF and IL-11, activates the JAK/STAT signaling pathway after SCIs. Yamauchi et al. demonstrated that peak IL-6 expression coincided with peak JAK1 and STAT3 activation as well as the nuclear translocation of phosphorylated STAT3 in neurons. The study shows that through the modulation of IL-6 and soluble IL-6 receptors, neurological functions can be improved, spinal motor neurons are protected, and neuronal degeneration is prevented (81). Mice with IL-6 knockout exhibit more severe injuries and spinal cord neuronal deaths (82). Further studies confirm the importance of IL- 6 in the regulation of the sensory function. Yamauchi’s study (83) demonstrated that through a pretreatment with the JAK2 inhibitor AG-490, limb functions were reduced after SCIs (84), suggesting that IL-6 might have protective effect on neurons after SCIs by increasing the activity of the JAK/STAT signaling pathway. As a potent trophic and pro-inflammatory factor, the concentration of LIF increased within 24h after SCIs, indicating its important role in modulating inflammatory responses and protecting oligodendrocytes after SCIs. Both NGF and CNTF affect the CNS in different ways, including differentiation and proliferation, but both of them support brain oligodendrocyte survival (85). Stirling et al. (86) have shown that CNTF receptors are upregulated in motor neurons in the anterior horn of the spinal cord and that CNTF is increased in the white matter 24h after SCIs. SCIs also induce reactive astroglial intracellular CNTF expression. In their study (87), Garcia-Alas G et al. noted that CNTF induced the rapid phosphorylation of JAKI, JAK2 and STAT1a/b, meanwhile the CNS cell survival increased via JAK-STATs.

## 3 JAK/STAT in homeostasis and SCIs

SCIs induce the negative regulation of P-STAT tyrosine in macrophages. The main roles of the JAK/STAT pathway include: (i) Microglia is activated after SCIs, M1-type microglia upregulates STAT1 after an INFγ+LPS/TNF-α stimulation to promote inflammation, eliminate debris, sterilize and remove apoptosis; M2-type cells induced by IL-4 and IL-13 upregulate STAT6 expression, thereby playing an anti-inflammatory role, promoting cell proliferation, migration, growth and myelin regeneration (88); (ii) Activated microglia after SCIs is highly expressed through the JAK/STAT3 pathway, thereby stimulating astrocyte proliferation; (iii) STAT3 plays an important role in astrocyte management and SCI-damaged areas, which is activated, and astrocytes will mediate the proliferation of scar tissues while promoting regeneration (89), the formation of which will limit the spread of inflammation (89); (iv) The differentiation direction of endogenous neural stem cells and neural precursor cells after SCIs is regulated through the JAK3 pathway, the over-activation of JAK3 drives the transformation of stem cells towards microglia, and the inhibition of the JAK pathway leads to a transformation towards oligodendrocytes as well as neurons (90); (v) After an exogenous stem cell transplantation, the JAK/STAT pathway synergizes with reactive astrocytes at the injuries, which works in concert with reactive astrocytes at the sites of injuries to promote axonal regeneration and suppress negative gene expression (91). Leukemia inhibitory factors and ciliary neurotrophic factors, both from the IL-6 superfamily, induce the differentiation of neural precursor cells into astrocytes via the JAK/STAT pathway (92). The increased IL-6 after SCIs promotes the differentiation of endogenous stem cells into astrocytes rather than oligodendrocytes or neurons (93), and the authors concluded that the increased differentiation of astroglial promoted glial scar formation, thereby inhibiting axonal regeneration after SCIs. Subsequent investigators found that the glial scars formed 2 to 3 weeks after SCIs were not the result of endogenous neural stem cell differentiation, but a structure formed by localized newly-proliferating glial cells (94). Some investigators suggest that IL-6 promotes axonal regeneration by promoting the upregulation of the expression of a nerve-growth-associated protein (GAP-43) and the downregulation of the axonal overgrowth inhibitor (Nogo)-A as well as receptor Nogo, thereby promoting axonal outgrowth and improving the motor function (93).

The Activation of the JAK/STAT Pathway and Changes in Downstream Factors after SCIs.

### 3.1 The activation of the JAK/STAT pathway after SCIs

SCIs are usually caused by traumas or partial diseases that severely affect the motor function of patients. In a study on STAT signaling in peripheral nerve injuries (95), the JAK1, JAK2, JAK3 and STAT1 mRNA increased instantaneously and rapidly after peripheral nerve injuries, meanwhile STAT3 and STAT5 gene expression was detected using the *in situ* hybridization and semi-quantitative polymerase chain reaction (PCR) technique through a regeneration study on the facial and hypoglossal nerves in rats. Only STAT3 protein consistently increased and was continuously activated. Peripheral nerve injuries simultaneously activate the JAK/STAT3 pathway in microglia on the dorsal side of spinal cord. The activation of STAT3 after SCIs plays an important regulatory role in astrocyte proliferation and glial scar formation. Kirshblum et al. also showed (96) that STAT3 played a critical regulatory role in the repair of injured tissues, the cellular recovery after SCIs and the recovery of the motor function. Through Western blot, a significant activation of the JAK1/STAT3 pathway in various cell types 6h after SCIs was confirmed (97). In the acute phase of SCIs, STAT3 phosphorylation is activated in spinal cord neurons, which contributes to neuronal protection; however, in the chronic phase, STAT3 phosphorylation is activated in microglia and astrocytes (89). Through immunofluorescence staining, the activation of p-STAT3 at the Tyr 705 locus is also confirmed with a series of changes in the expression of STAT1 activation after ischemia-reperfusion injuries exacerbated by SCIs. However, the temporal or spatial alterations of STATE after SCIs have not been fully elucidated.

The JAK/STAT pathway was originally discovered in the interferon γ signaling system (98–100). Interferon γ, a pro-inflammatory cytokine, has been shown important in controlling phosphorylation and STAT1 nuclear translocation as well as modulating the expression of major histocompatibility complex (MHC) genes. Interferon γ appeared in the cytoplasm of motor neurons 2 days after neurotomy and remained there for 8 days, suggesting that it may be involved in the process of nerve regeneration. After SCIs, neutrophils infiltrate the lesion sites, causing an early spinal cord inflammatory response. These neutrophils are capable of releasing reactive oxygen species, together with cytokines and chemokines. A severe oxidative stress is induced after SCIs. Minghetti L et al. (101) reported superoxide dismutase production 6h after SCIs, along with an increased mitochondrial cytochrome C release and caspase-9 cleavage, as well as motor neuron deaths in the ventral anterior horn of the spinal cord 1 day after SCIs. SCIs were improved in superoxide-dismutase-overexpressing rats, and reactive oxygen species also contributed to spinal motor neuron damage after SCIs. CNS neurons are the most sensitive cells to oxidative stress, and oxidative stress injuries act in several diseases, including Parkinson’s and Alzheimer’s. In addition, the oxidative stress level of patients with amyotrophic lateral sclerosis increases and such oxidative stress is accompanied by the activation of the JAK/STAT pathway. It is reported in previous studies (102) that in the basilar artery of the subarachnoid space, oxidative stress activates STAT1 and the JAK/STATI pathway, which may be associated with apoptosis after SCIs.

### 3.2 Correlation of the JAK/STAT signaling pathway with inflammatory factors

Inflammation is a major process secondary to injuries that directly contributes to both acute and chronic SCIs, which is critical for causing neurological damage. At the same time, inflammatory responses may lead to the deaths of neurons and oligodendrocytes, as well as the formation of palsy scars, ultimately causing the loss or reduction of neurological functions; therefore, it is believed that reducing inflammatory responses can help reduce secondary SCIs and the functional deficits after SCIs.

After SCIs, blood vessels rupture, disturbing the blood-brain barrier, and neutrophils rapidly infiltrate in the injury sites. There is a possibility that secondary spinal cord damage results from this process (56). The presence of TNF-positive cells in spinal cord segments can be observed 30 to 45min after SCIs, and the cells persist for 3 to 24h. TNF- a and IL-6 are rapidly upregulated in the SCI areas. Inflammatory cytokine mRNA expression was found 15min after SCIs in rats, with TNF-a increasing first, followed by IL-6 mRNA. In rat spinal cord tumors, TNF-a is able to enhance glutamate-mediated neuronal cell deaths, while TNF antagonists are able to reduce inflammatory responses and tissue damage. Additionally, monoclonal antibodies targeting at the IL-6 receptor make astrocyte differentiation, inflammatory cells and connective tissue scar formation reduced. According to experiments, through IL-6 infusion, the neutrophil number increases by six-fold with that of macrophages by two-fold, microglia is activated and the axonal growth at injured locations is reduced. Studies on the IL-1 family (56, 101, 102), such as IL-1, have shown the ability to exacerbate the inflammatory response process induced by SCIs. Both IL-1 and IL-18 are potent inflammatory mediators capable of provoking and/or intensifying a wide range of innate immune-related responses, such as tissue damage and host responses to microbial invasion. In addition, it is hypothesized that in normal tissues, Caspase-1 is blocked by inhibitors, which remains inactive, and studies have shown that in normal rat spinal cord, with the presence of a protein component consisting of neutrophil alkaline phosphatase 1 (NALP1), adipose-derived stem cells (ASCs), Caspase-1 and Caspase-2 with inhibitors of members of the apoptosis suppressor family, X apoptosis inhibitory protein (XIAP), SCIs cause IL-1 and IL-18 expression, activate Caspase-1, cleave XIAP, and promote NALP1 assembly. Also, ASC reduces Caspase-1 activation and XIAP cleavage, as well as IL-1 and IL-18 expression, which lead to the reduction of pathological changes and functional impairments in spinal cord tissues.

After CNS injuries are initiated by peripheral immune cells, with glial cells proliferating or migrating to the lesion sites. T-cell activation is essential for macrophage and immune responses. The activation of reactive T cells of myelin basic protein (MBP) by SCIs in rats induces neuroinflammatory responses and increases the frequency of MBP-reactive T cells in SCIs after transient nerve paralysis to a level approximating that of patients with multiple scleroses. Other experiments have also confirmed a direct link between lymphocyte activation and primary CNS pathology. Once entering the injured sites, lymphocytes persist indefinitely (103). An increase in the number of T. B cells can be found 9 weeks after SCIs in rats (104). It is hypothesized that macrophages and neutrophils are also involved in tissue destruction and injury expansion. Macrophages and microglia contribute to secondary inflammatory responses through the release of TNF-Q, IL-1, IL-6 and IL-10 through cytokines, interferons and interleukin receptors (IL-4R and IL-2R, etc.). Cytokines trigger inflammatory responses of the central nervous system by triggering the expression of other cytokines, chemokines and reactive oxygen species.

Previous reports (56, 101, 103, 105) show that inflammatory cytokines such as TNF-a, IL-1B and IL-6, etc. after SCIs can all exacerbate secondary SCIs, and the significance of an upregulated TNF-a, IL-1 and IL-6 gene expression after SCIs starts rapidly 3-6h after SCIs. In some studies, researchers O’Shea et al. (106) tried to reduce SCIs by inhibiting the expression of these genes. In cytokine responses, the JAK/STAT pathway is an important transduction pathway from the cell surface to the nucleus. Binding of cytokines to intracellular receptors at the cell membrane induces glycoprotein (GP) dimerization, followed by JAK activation at the cell membrane, STAT1 phosphorylation at the cytoplasm and signals from phosphorylated STAT1 into the nucleus, causing the transcription of target genes, a pathway that plays an important role in pathophysiological changes following SCIs. It was shown that microglia- and macrophage-mediated immune responses after SCIs exacerbated secondary spinal cord 24h after SCIs, meanwhile TNF-a, IL-1 and IL-6 increased substantially, which aggravated secondary SCIs. STAT1 inhibition blocks the action of these pro-inflammatory factors. STAT1 gene silencing results in a significant increase in IL-10, which provides direct neuroprotection, and TL-10 can be used as an effective adjunctive therapy for SCIs. Via selective STAT1 inhibition through different modalities or STAT1 gene deletion, the release and expression of the above-mentioned inflammatory factors can be reduced, thereby reducing secondary damage to spinal cord and protecting its neurological functions.

Studies have reported the activation of the neuronal JAK/STAT signaling pathway after cerebral ischemia, in which STAT3 plays an important role in neuroprotection by inducing neuroprotective genes such as Bc1-2, with an increasing STAT3 expression in ischemic regions. Previous studies have shown that the STAT3 signaling pathway promotes neuronal survival (107). The inhibition of IL-6 decreases STAT3 phosphorylation, which leads to the exacerbation of cerebral infarction. After myocardial ischemia, STAT3 activation promotes cardiomyocyte survival. Mice defective in the STAT3 gene exhibit increased infarct foci areas. In inducible nitric oxide synthase (iNOS)- and cyclooxygenase (COX-2)-pretreated myocardial ischemic mice, the activation of the JAK/STAT3 signaling pathway plays an important role in ischemia-reperfusion injuries, as is evidenced by an enhanced ischemic tolerance (36). The JAK1/STAT3 pathway plays a critical role in neuronal protection after SCIs in motor neurons. Ischemic-injury-phosphorylated STAT3 translocates into the nucleus and stimulates the transcription of target genes, thus acting as a neuroprotective agent. Amino acid supplementation activates STAT1 and STAT3 in ischemic myocardium, and it is found that protective STATS is preferentially activated over apoptotic STAT1, resulting in improved myocardial cell ischemia (36, 108). The granulocyte colony-stimulating factor (G-CSF) induces the activation of STAT1 and STATE in cardiomyocytes, and although STAT3 is preferentially activated, the balance between the protective function of STAT3 and the apoptotic function of STAT1 determines the fate of the cells. Through ischemia-reperfusion injuries in endothelial cells PI3K/Akt and p38 mitogen-activated protein, protein kinase (a MAPK-dependent pathway, CCO monoxide) decreases the expression of phosphorylated STAT1 and increases the expression of phosphorylated STAT3, leading to endothelial cell survival (109–111). STAT is transferred to the nucleus after tyrosine phosphorylation, where it is phosphorylated through the p38 MAPK pathway at the serine Ser727 site. The phosphorylation of STAT1 at its Ser727 site results in the apoptosis of cardiomyocytes with ischemia-reperfusion injuries. Tetramethylquatha tempol (4-hydroxy-2, 2, 6, 6-tet-ramethylpiperidine-i-oxyl), a free radical scavenger, protects myocardium and ameliorates ischemic perfusion injuries in cardiac myocytes by inhibiting the acid phosphorylation of STAT1 at the Ser727 site. Immunoblotting and immunofluorescence data shows that STAT1 phosphorylation at the Tyr 701 site is in the cytoplasm, but not the nucleus, together with p-STAT1, which is induced into the nucleus. In the case of severe injuries, the phosphorylation of the STAT1Ser727 site leads to apoptosis, whereas that of STAT3 causes intranuclear signaling with anti-apoptotic effect (112).

Oxidative damage is the etiology of many diseases, including Parkinson’s and Alzheimer’s disease, meanwhile lateral spinal cord sclerosis and these oxidative stresses are accompanied by the activation of the JAK/STAT pathway. It has also been reported that oxidative stress is accompanied by an oxidative stress response with the activation of STAT1 in subarachnoid hemorrhage. These studies suggest that the JAK1/STAT1 signaling pathway may induce neuronal deaths after related SCIs. The JAK1/STAT3 pathway is activated after cerebral ischemia. STAT3 plays a critical role in neuroprotection through the neuroprotective gene bc1-2, and the inhibition of STAT3 can result in the increase of cerebral infarction (113, 114). Previous studies suggest that: the inhibition of IL-6 leads to a decreased STAT3 phosphorylation, which exacerbates cerebral ischemia, and the activation of STAT3 increases ischemic myocardial survival. During myocardial ischemia-reperfusion injuries, STAT3-deficient rats have larger cerebral infarct sizes and lower cardiac functions (114). The JAK 1/STAT3 activation contributes to neuroprotection. Both STAT1 and STAT3 are activated in the anterior horn neurons of the spinal cord after mild SCIs, with STAT3 phosphorylated at the Tyr 705 site in the nucleus of neurons 6h after SCIs and STAT1 phosphorylated at the Tyr 701 site in the cytoplasm of neurons 6h after SCIs, and it is STAT3 but not STAT1 that conducts signals to the nucleus in the anterior horn neurons of the spinal cord, leading to gene transcription.

The sustained activation of the Janus kinase/signal transducer and the activator of the transcription (JAK/STAT) signaling pathway are closely related to many immune diseases, inflammation and tumors (115–117), and different STAT proteins are highly specific in the process of cytokine signaling. Different STAT proteins are highly specific in their involvement in cytokine signaling. Signal transducer and activator of transcription (STAT) regulate CD4+ T cell division towards Th1 (118), those of transcription 6 (STAT6) regulate the differentiation of CD4+ T cells towards Th2 (119), meanwhile those of transcription 4 (STAT4) and STAT6 mediate cytokine transcription, all of which are through the JAK/STAT signaling pathway.

In addition, transcription factors are also one of the important factors affecting CD4+ T cell differentiation, in addition, the phosphorylation of STAT transcription factors and their transduction to the nucleus are important steps in regulating the balance of Th1/Th2 cytokines (120, 121). Studies have shown (122–124) that the JAK/STAT signaling pathway is closely related to CD4+ T lymphocyte differentiation. Therefore, JAK/STAT-signaling-pathway-related assays can reflect the functional immune response status of both *in vivo* and *in vitro* lymphocyte subpopulations to a certain extent (125).

## 4 The regulation of JAK/STAT signaling pathway

The positive regulation mechanism of JAK/STAT is mediated through the binding of 6 cytokines, namely interferons (INFs), growth factors (GFs), colony-stimulating factors (CSFs), interleukin, tumor necrosis factors (TNFs) and chemokines (CFs), to their corresponding receptors, meanwhile in the FERM region of JAK2, the cytokine receptor proximal membrane region motifs are recognized and the oligomerization of receptors is induced. These phosphorylated tyrosines are used as anchor points to recruit various signaling molecules containing specific SH2 structural domains (e.g., STATs), which are activated to form dimers into the nucleus to exert transcription factor activity while promoting the transcription and translation of growth and proliferation-related genes, resulting in an upregulation of expression.

**Figure 3:**
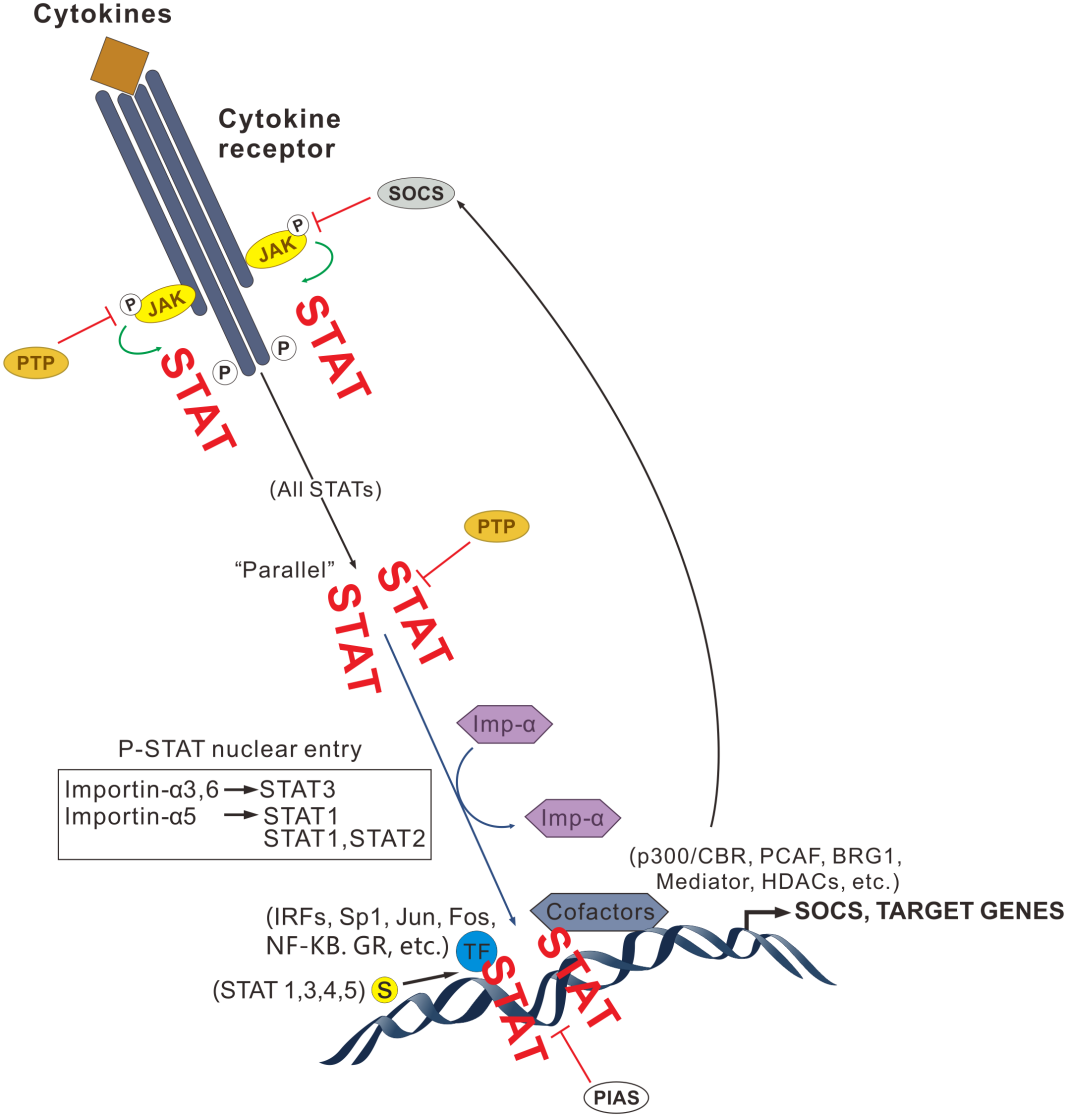
JAK/STAT signaling pathway regulation.

The negative regulation of JAK/STAT mainly involves 3 negative regulators: suppressors of cytokine signaling (SOCSs), protein inhibitors of activated stats (PIASs) and protein tyrosine phosphatases (PTPs). In addition, there are many negative proteins, such as PTPs. The SOCS family (also known as the CIS or SSI family) consists of at least 8 proteins: SOCS1 to SOCS7 and CIS, all of which have an intermediate SH2. SOCS is induced to activate by a wide range of cytokines, such as IL-1, IL-2, IFN, erythropoietin (EPO), G-CSF and GM-CSF, etc, which inhibit the activation of JAKs by binding to their tyrosine phosphorylation sites, which inhibits the activation of their downstream signaling molecules by competing with STATs, and negatively regulate cytokine signaling by promoting the proteasomal hydrolysis of JAK/STAT pathway signaling proteins. Studies have shown that SOCS1 inhibits the enzymatic activity of phosphorylated JAK2 by directly binding to it, and that SOCS3 inhibits JAK activation by binding to the activated receptors (126). The SOCS-1 gene expression was observed in RA through immunohistochemistry, and SOCS-1 gene was mainly expressed by macrophages, lymphocytes as well as fibroblasts in synovial tissues, meanwhile an enhanced synovial cell infiltration, enlarged lymphocytes and highly-proliferated T cells were observed in SOCS-1-/-IFN-/- double-knockout arthritic rats. Shouda et al. (127) used Northern blot to detect the expression of CIS3 in RA and OA. The results showed that CIS3 was strongly expressed in RA, while in OA, CIS3 expression was significantly weaker than that in RA. Adenoviruses carrying recombinant CIS3DNA were also injected into the ankle joints of antigenic and collagen-induced arthritic rats respectively. SOCS negatively regulates JAK-mediated gene expression in a negative feedback loop (128): activated JAKs activate downstream signals such as STATs while inducing SOCS expression; the expression products of SOCSs, in turn, specifically regulate SOCS expression, whose expression products, in turn, specifically inhibit this JAK signaling pathway. The second repressor of the JAK/STAT pathway is Bcl-6, which acts as a transcriptional repressor mainly by binding to STAT6. The third negative regulatory site is the pseudokinase, the JH2 structural domain of JAK proteins. PTPs can also negatively regulate JAK/STAT, for example, the SH2-structural-domain-containing PTP1 (SHP1, SHP2) and CD45 as well as the activated STAT nuclear protein repressors (PIASs) (**Figure 3**).

## 5 JAK/STAT inhibition mechanism

### 5.1 JAK/STAT and lymphocyte differentiation

JAK/STAT receptor-mediated activation leads to T cell differentiation in thymic or inflammatory loci (129). In a similar pattern, IL-4 and IL-6 affect the preferential conversion of Th2, but IL-12 affects the preferential conversion of Th1. In addition, IL-6 converts monocytes to macrophages. In many types of immune and autoimmune diseases, the dysregulation of cytokines often leads to inflammatory responses. Excess Th1 (opposed to Th2) in pancreatic islets is often considered a precursor of islet inflammation. A potent pyrazole-pyrimidine (PP) inhibitor, PP1, increases the expression of Th1 in spleen cells from ovalbumin-specific T cell receptor (TCR)-transgenic BALB/c mice while culturing PP1, acting through the direct inhibition of Lck, Fyn or Hck kinases, which are involved in TCR cell signaling and subsequent T cell activation. Lck kinase phosphorylates a multicomponent region of cells during the synergistic stimulation of T cell activation with antigen-presenting cells (APCs). Thus, PP1, its isoforms and molecules with the same activity, are potential therapeutic agents for autoimmune diseases.

### 5.2 Immune rejection of JAK/STAT and allografts

T cells respond to allogeneic grafts at specific sites (130). The responses of lymphocytes are regulated by a complex set of interactions between receptors and ligands. Acute immune rejection is chemotactic due to cytokines (e.g., IL-2). The allosteric signal transduction block induced by anti-IL-2Rα monoclonal antibody administration demonstrates tolerance to alloantigens. However, IL-10 is a highly-efficient immunosuppressive ligand, affecting the functions of T cells, B cells and APCs. Typically, IL-10 downregulates immune responses in the same pattern as IL-6 via JAK/STAT and various SOCS molecules. Recently, a variant of the antibiotic undecylenic mycoerythrin family, PNU156804, was found to prolong the graft lifespan of synergy with cyclosporine A inhibitors; such effect was increased by blocking graft rejection through JAK3 targets in synergy with sirolimus (also known as rapamycin). In addition, FK778, applied alone, prolongs the graft lifespan by inhibiting JAK3. Although cytokines and JAKs are involved in immune rejection, the extent of inhibition, specific target sites and the specific roles of individual JAKs need to be further investigated.

## 6 Conclusion

Molecular interactions between JAKs and STATs mediate cellular responses that play an important role in both neural homeostasis and neuroinflammation. Molecules that interfere with these interactions, particularly small molecules targeting at JAKs and STATs, have shown a good efficacy and safety in animal studies for the treatment of SCIs. In contrast to other therapies for SCIs, JAK inhibitors are orally bioavailable with predictable pharmacokinetics, which are not immunogenic. Although STAT inhibition may be an alternative therapeutic route for targeting inflammation, no clinical trials have been conducted to date for the treatment of SCIs. As more drugs enter the clinical development and SCI treatment devices, and as technologies advance, the optimization of therapies requires a deeper understanding of disease mechanisms and drug modes of action to support patient selection and treatment stratification. This approach, also known as “precision” or “personalized” medicine, has received significant attention in the past decade. However, over time, the mechanisms that drive disease heterogeneity among and within patients have remained poorly-understood, which greatly limits our ability to predict the best therapies for patients or responses to those therapies. We believe that an understanding of disease heterogeneity is needed with the goal of identifying the key molecular and cellular patterns that drive individual disease pathology and behavior, so as to predict the best therapeutic approaches. To achieve this goal, longitudinal studies in which higher-resolution methods (e.g., single-cell transcriptomics) are applied to the analysis of SCI samples may be required.

## Author contributions

XG: Writing – original draft. CJ: Conceptualization, Data curation, Writing – review & editing. ZC: Formal Analysis, Writing – review & editing. XW: Visualization, Writing – review & editing. FH: Formal Analysis, Visualization, Writing – review & editing. DH: Writing – review & editing.
